# The KIF13A-Rab11a axis: a key regulator of vesicle trafficking in cytokinesis

**DOI:** 10.17179/excli2026-9394

**Published:** 2026-06-19

**Authors:** Paulius Gibieža, Sergi Rodriguez-Calado, Vilma Petrikaite

**Affiliations:** 1 Institute of Biotechnology, Life Sciences Center, Vilnius University, Saulėtekio Ave. 7, LT-10257, Vilnius, Lithuania; 2 Laboratory of Drug Targets Histopathology, Institute of Cardiology, Lithuanian University of Health Sciences, Sukilėlių Ave. 13, LT-50162, Kaunas, Lithuania; 3 Cell Division and Cytoskeleton, Danish Cancer Institute, Strandboulevarden 49, 2100, Copenhagen, Denmark

**Keywords:** KIF13A, Rab11a, transport, cytokinesis, division

## Abstract

Cytokinesis requires tightly coordinated membrane trafficking and cytoskeletal remodelling to ensure accurate physical separation of daughter cells. Rab11a, a recycling endosome-associated small GTPase, and KIF13A, a kinesin-3 family microtubule motor protein, have each been implicated in late mitotic events. However, the mechanistic relationship between Rab11a-mediated endosomal trafficking and KIF13A-driven transport during abscission remains insufficiently defined. This study aimed to elucidate the spatial and functional interplay between Rab11a and KIF13A during cytokinesis and to determine the consequences of disrupting this axis on mitotic progression and genomic stability. Rab11a and KIF13A were depleted in HeLa cells using shRNA- and siRNA-mediated knockdown strategies. Subcellular localisation and colocalisation at the intercellular bridge were examined by immunofluorescence microscopy and quantified using Pearson's correlation coefficient. Functional outcomes were assessed through multinucleation, micronuclei formation, telophase accumulation, and LAP2-positive chromatin bridge assays. Mitotic timing from nuclear envelope breakdown to abscission was quantified by live-cell spinning-disk confocal microscopy. Rab11a and KIF13A exhibited pronounced colocalisation at the ICB during cytokinesis. Depletion of either protein reduced its spatial association with the other. It resulted in marked cytokinetic defects, including increased multinucleation, micronuclei formation, telophase delay, and elevated chromatin bridge frequency. Live-cell imaging demonstrated a significant prolongation of the cytokinesis-to-abscission interval, accompanied by an extended overall mitotic M-phase. Mechanistically, loss of Rab11a or KIF13A impaired CHMP4B recruitment to the ICB, reduced PI3P accumulation, and enhanced Aurora B signalling, consistent with activation of the abscission checkpoint. These findings establish the Rab11a-KIF13A axis as a critical regulator of late mitosis, integrating endosomal trafficking with ESCRT-III-dependent abscission. Disruption of this pathway compromises abscission accuracy and promotes genomic instability, highlighting its essential role in maintaining mitotic integrity and its potential relevance in tumorigenesis. Continued exploration of the KIF13A-Rab11 interaction will yield a deeper understanding of mitotic progression and cellular organisation.

## Introduction

Rab proteins are small monomeric GTPases that form the largest subgroup within the Ras GTPase superfamily. These proteins are evolutionarily conserved and play crucial roles in membrane trafficking across eukaryotic cells. In humans, there are approximately 70 distinct Rab GTPases, each associated with specific intracellular membranes based on their functional roles (Li and Marlin, 2015[28]). Rab proteins operate through a cycle between an active, GTP-bound state and an inactive, GDP-bound state. This dynamic transition is regulated by guanine nucleotide exchange factors (GEFs), which activate Rabs, and GTPase-activating proteins (GAPs), which inactivate them (Stenmark, 2009[42]; Homma et al., 2021[22]). In their active state, Rabs localise to specific organelles or vesicles, where they recruit a range of effector proteins that mediate various aspects of membrane transport. Identifying their effectors has been key to understanding their specific cellular functions (Hutagalung and Novick, 2011[23]). Rab effectors are diverse and include vesicle tethers, kinases, phosphatases, adaptors, and motor proteins (Grosshans et al., 2006[18]). Through these interactions, Rabs orchestrate the four principal stages of membrane trafficking: vesicle budding, transport, tethering, and fusion with target membranes (Novick, 2016[34]). As such, Rab GTPases are crucial for maintaining essential physiological processes, including hormone secretion, neurotransmission, immune responses, and overall cellular homeostasis. Dysregulation of Rab expression or function is implicated in numerous human diseases, including neurodegenerative diseases (such as Alzheimer's and Parkinson's), infections, immune abnormalities, and cancer (Guadagno and Progida, 2019[19]).

Given the essential role of Rab proteins in membrane trafficking and cellular homeostasis, their functions in signalling and membrane trafficking during cell division are equally critical. One of the pivotal contributions of Rab GTPases is to ensure the successful completion of cytokinesis and abscission in the final phases of cancer cell division. Disruption of these processes can delay abscission, leading to incomplete cell separation and the potential formation of multinucleated cells (Wilson et al., 2005[49]). Among the best-characterised Rabs in this context is Rab11, which plays a central role in coordinating membrane trafficking during mitosis. Rab11-positive endosomes have been shown to associate with mitotic spindle poles and the mitotic spindle, thereby contributing to proper mitotic progression (Hehnly and Doxsey, 2014[21]). Our previous findings, consistent with those of other laboratories, demonstrate that Rab11 is crucial for regulating cancer cell division by mediating the targeted delivery of cytoplasmic membranes to the cleavage furrow, intercellular bridge (ICB), and midbody (MB) (Collins et al., 2012[8]; Gibieža et al., 2025[16]). These Rab11-enriched vesicles contribute to membrane constriction at the cleavage site, essential for furrow ingression during late anaphase. In addition, Rab11 and its effectors, such as Rab11 family-interacting protein 3 (FIP3) and Rab11 family-interacting protein 1 (FIP1), are associated with recycling endosomes that traffic to the cleavage furrow or are recruited to the MB during cytokinesis (Wilson et al., 2005[49]; Collins et al., 2012[8]). At the ICB, the Rab11/FIP1 complex facilitates the localisation of Rab35, another Rab family member, which promotes actin disassembly and abscission. This complex also ensures proper recruitment and polymerisation of the endosomal sorting complex required for transport (ESCRT) near the MB (Iannantuono and Emery, 2021[24]), a key step that initiates actin-dependent microtubule severing and enables the final separation of the daughter cells (Advedissian et al., 2024[2]).

For the reasons stated above, it is evident that the efficient transport of Rab11-positive vesicles to the abscission site is crucial in preventing cytokinesis failure, which can lead to aneuploidy and promote tumour progression in cancer cells (Wilson et al., 2005[49]). However, the mechanisms by which Rab11-positive endosomes traverse the cytoplasm along the extended cytoskeletal network to reach the abscission site and the MB at the ICB remain poorly understood. It is plausible that some molecular motors facilitate the directed movement of these recycling endosomes, which are required for the final separation of dividing daughter cells. In this context, a study by Fielding et al. demonstrated that Rab11/FIP3-positive endosomes are transported along central spindle microtubules toward the ICB in a Kinesin-1-dependent manner (Fielding et al., 2005[15]). The kinesin superfamily (KIF) comprises ATP-dependent motor proteins that transport various cargoes, including vesicles, along the tracks of the microtubule network. To date, approximately 45 KIF proteins have been described. They are categorised into 14 subfamilies based on their sequence homology and domain architecture. These motor proteins support numerous cellular processes, such as vesicle transport, cell division, and microtubule cytoskeleton remodelling. As an example, the studies by Sagona et al. and Montagnac and Chavrier identified KIF13A-a kinesin-3 family motor protein-as a key regulator of intracellular transport for various signalling molecules during cytokinesis (Montagnac and Chavrier, 2010[31]; Sagona et al., 2010[38]).

Until now, not without a reason, KIF13A has been shown to influence the morphogenesis of recycling endosome tubules and membrane dynamics, affecting their length and abundance. This motor function was shown to be critical for diverse cellular processes, including cargo transport, establishment of cell polarity, migration, and cytokinesis (Thankachan and Setty, 2022[43]). KIF13A has been recognised as a kinesin of the recycling endosome, primarily based on its association with Rab11a (Delevoye et al., 2014[12]; Thankachan and Setty 2022[43]). Indeed, KIF13A has been shown to interact with all three isoforms of Rab11, including Rab11a, Rab11b, and Rab25, through yeast two-hybrid assays and pull-down experiments (Delevoye et al., 2009[11], 2014[12]). Therefore, this plus-end-directed kinesin-3 motor protein is a strong candidate for mediating the transport of Rab11-positive vesicles. Although direct studies examining the KIF13A-Rab11 interaction specifically during cell division are limited, several lines of evidence suggest functional and transport-related connections between the two proteins (Delevoye et al., 2014[12]; Ramos-Nascimento et al., 2017[36]). These studies emphasise both the individual and cooperative roles of Rab11 and KIF13A in regulating endosomal dynamics and cell division processes. Notably, Rab11 and KIF13A have been shown to act together to form recycling endosomal tubules originating from sorting endosomes. These tubules travel along microtubules toward their plus ends, directed to the ICB (Delevoye et al., 2014[12]). This interaction likely occurs at specialised subdomains of sorting endosomes, where Rab11 and KIF13A collaborate to generate recycling endosome tubule intermediates (Delevoye et al., 2014[12]). In the context of recycling endosome morphogenesis in HeLa cells, a study by Delevoye et al. demonstrated that depletion of KIF13A disrupts the formation of Rab11a-positive recycling endosomes, resulting in the generation of enlarged endosomes (Delevoye et al., 2014[12]).

To date, the mechanism of KIF13A action during cell division has been well-characterised. KIF13A interacts with FYVE-CENT and TTC19 to form a complex that travels along microtubules in a plus-end-directed manner, targeting Phosphatidylinositol 3-phosphate (PI3P) at the MB. This interaction leads to the activation of charged multivesicular body protein 4B (CHMP4B), a core component of the ESCRT-III complex. In turn, CHMP4B triggers a signalling cascade that promotes actin clearance and initiates microtubule severing at the ICB, ultimately enabling the successful physical separation of the two daughter cells (Montagnac and Chavrier, 2010[31]; Sagona et al., 2010[38]). Building on this, KIF13A was initially identified by several research groups as a microtubule motor protein that mediates the transport of various vesicles and endosomes (Nakagawa et al., 2000[33]; Delevoye et al., 2009[11]). These findings confirm that the KIF13A-FYVE-CENT-TTC19 complex regulates the trafficking of PI3P-positive endosomes, which is essential for sustaining recycling within the ICB, and thus controlling the vital factors required for cytokinesis. Notably, a few other studies have demonstrated an intriguing observation that TTC19 associates with ESCRT complex components, which are implicated in the abscission mechanism (Carlton and Martin-Serrano, 2007[7]; Morita et al., 2007[32]). Thus, a theory was proposed that the ESCRT machinery may contribute to either the trafficking of endocytic structures to the ICB or to the cleavage of the ICB. Nevertheless, the data from the Hanson et al. laboratory suggest that ESCRT protein complexes induce membrane deformations that occur during abscission, thereby regulating the final cleavage of the ICB (Hanson et al., 2008[20]).

Based on current evidence, we propose that KIF13A may facilitate the targeted transport of Rab11-positive vesicles from recycling endosomes to the MB and abscission site at the ICB. These vesicles can subsequently interact with PI3P, triggering the ESCRT-III-mediated membrane fission cascade that drives the final steps of cytokinetic abscission. To test this hypothesis, we first aimed to determine the localisation of the motor protein KIF13A and its putative cargo Rab11a, along with other downstream binding partners of the PI3P-mediated abscission cascade, during cytokinesis in cells treated with either a non-targeting-shRNA control or different shRNAs separately targeting Rab11a and KIF13A. This helped us to generate an initial insight into the interrelation between the two. After determining their localisation, we next assessed the colocalisation of KIF13A and Rab11a under the conditions described above, as this would help to reveal whether these proteins interact or function together spatially during cell division. Next, to explore their involvement in cytokinesis regulation, we conducted several functional assays. The multinucleation and micronuclei assays enabled us to evaluate the functional roles of Rab11a and KIF13A in the cytokinesis process. In addition to the aberrant cleavage furrow formation observed upon KIF13A and Rab11a depletion, a small part of the cells were also arrested or delayed in telophase, indicating impaired progression from telophase to the completion of cytokinesis. Moreover, downregulation of Rab11a or KIF13A resulted in the formation of chromatin bridges within the mitotic cell population. In addition, it increased the time required for cell division, as observed through time-lapse imaging. Overall, the cytokinesis defects observed in Rab11a- and KIF13A-depleted cells likely reflect the disruption of targeting Rab11-positive endosomal tubules to the MB and the abscission sites on the ICB. Investigating the KIF13A-Rab11 axis may therefore provide new insights into abnormalities caused by aberrations in Rab11a trafficking, which eventually could lead to increased tumorigenesis in cancer cells.

## Methods

### Cell culture

The human adenocarcinoma HeLa-wt cell line was obtained from the American Type Culture Collection (ATCC, Manassas, USA). HeLa GFP-Tubulin mCherry-H2B cells were provided as a kind gift by Ulrike Gruneberg (University of Oxford (Zeng et al., 2010[51])). Both cell lines were maintained in 5 % CO_2_ and at 37 °C in Dulbecco's modified Eagle's medium (DMEM, Life Technologies Gibco, USA), supplemented with 10 % fetal bovine serum (Life Technologies Gibco, USA) and a 1 % solution of 10,000 units/ml penicillin and 10 mg/ml streptomycin (SIGMA, USA). Cells were regularly tested for Mycoplasma contamination. 

### Generation of lentiviral stable cell lines

Lentiviral transduction particles were acquired from Sigma Aldrich (USA). Target sequences used for shRNA treatment are as follows: Non-targeting shRNA control (#SHC016V-1EA), Rab11a-KD1 - 5'-GCCTTATTGGTTTATGACATT-3' (#TRCN0000073022), Rab11a-KD2 - 5'-AGTTGTCCTTATTGGAGATTC-3' (#TRCN0000381243), KIF13A-KD1 - 5'-GGAAACCTCCCAAGGTATTTG-3' (#TRCN0000364983), and KIF13A-KD2 - 5'-TTAACGAACTTCTGGTTTATT-3' (#TRCN0000377368). Different lentiviral particles mixed with media were pre-treated with polybrene (Merck Millipore, USA, #TR-1003-G) before being applied overnight to 50 % confluent target HeLa-wt cells. Cells were allowed to recover for 24 hours, then selected with puromycin (2 µg/ml), expanded and frozen as stocks. Low-passage cells were used in all experiments to ensure experimental consistency and reproducibility. The remaining expression of Rab genes of interest in cell lines was then validated using Western Blotting.

### siRNA treatments

HeLa cells stably expressing GFP-tubulin and mCherry-H2B were transfected with various siRNAs using RNAiMAX Reverse Transfection Lipofectamine (Thermo Fisher Scientific, USA, #13778075) according to the manufacturer's instructions (Thermo Fisher Scientific, USA). Briefly, cells were incubated with either 25 nM or 50 nM siRNA concentrations for 48 or 72 hours, allowing the siRNA to take effect before lysate collection for Western blot analysis. For time-lapse imaging, cells were treated with siRNA for 48 hours before the experiment. The double-stranded RNA sequences used for siRNA treatments were as follows: Silencer Select Negative Control #1 siRNA (Ambion, USA; #4390843), Rab11a siRNA1 - 5'-CAACAAUGUGGUUCCUAUUtt-3' (Ambion, USA; #4390824), Rab11a siRNA2 - 5'-AATGTCAGACAGACGCGAAAA-3' (Merck, USA; Custom siRNA in Tubes), KIF13A siRNA1 - 5'-CTGGCGGGTAGCGAAAGAGTA-3' (Merck, USA; Custom siRNA in Tubes), and KIF13A siRNA2 - 5'-CCGCAACAACTTGGTAGGAAA-3' (Merck, USA; Custom siRNA in Tubes). Each siRNA transfection was performed in at least three independent biological replicates. 

### Western blotting

For Western blot analysis, cells were scraped in 1× PBS containing 1 mM PMSF (Thermo Fisher Scientific, USA, #36978) and 1 % Triton X-100 (Thermo Fisher Scientific, USA, #A160046.AE), and incubated for 30 min on ice. The collected cells were centrifuged at 15,000 g for 5 min at 4 °C to pellet the lysates. The Bradford assay was used to determine the total protein (Thermo Fisher Scientific, Pierce, USA, #23238). Protein samples were mixed with 5× SDS sample loading buffer (Thermo Fisher Scientific, Pierce, USA, #39000) and boiled for 5 min at 95 °C. Based on molecular weight, proteins were resolved on 12 % SDS-PAGE gels, transferred to PVDF membranes, and probed with the appropriate primary antibodies following standard protocols. The expression of each protein was normalised to β-tubulin, used as the internal loading control. Protein quantification was performed through densitometric analysis with ImageJ software (National Institutes of Health, USA). Unless otherwise specified, all data are derived from at least three independent experiments (biological replicates), with the mean and standard deviation calculated. Asterisks (*) indicate statistically significant differences (p < 0.05). 

### Immunofluorescence staining

For immunofluorescence analysis, cells plated on collagen I-coated (at a final concentration of 50 µg/ml, Life Technologies Gibco, USA) No. 1 microscope cover glasses (VWR) were fixed using 4 % Paraformaldehyde (PFA) for 15 min at room temperature (R.T.). Cells were then quenched for 5 min with a Quench buffer (375 mg of glycine diluted in 1× PBS), permeabilised and blocked in the incubation buffer (1 % BSA, 1 % Saponin, 2 % FBS, all mixed in 1× PBS) for 30 min, at R.T. Next, the cover glasses were incubated with primary antibodies for 1 hour at 37 °C, in a moisture-maintaining incubator. Then, the cover glasses were incubated with secondary antibodies for 30 min at 37 °C, in a moisture-maintained incubator. Finally, the cover glasses were incubated with 1 µg/ml DAPI (Thermo Fisher Scientific, USA, #D1306) solution for 5 min, at R.T. Lastly, the cover glasses were mounted on microscopic slides with a Prolong Diamond Antifade mounting media (Invitrogen, USA, #P36965). 

Immunofluorescence was detected using an inverted fluorescence Olympus IX73 microscope (Olympus Europe Holding GmbH) using a 20× lens for multinucleation and mitotic stage analysis assays. An upright confocal Olympus Fluoview FV1000 microscope (Olympus Europe Holding GmbH) utilises a 20×, 40×, and 60× oil immersion lens for localisation, LAP2 chromatin bridge, and lagging chromatin bridge assays, depending on the requirements. Three biological repeats were completed for each data set, each biological repeat containing 5 to 10 technical replicates. Data are reported as the mean with the corresponding standard deviation.

### Live-cell imaging

To characterize the effects of Rab11a and KIF13A depletion during cell division, spinning-disk confocal live-cell time-lapse imaging was performed using a Plan-Apochromat DIC 63x/1.4 NA oil objective with differential interference contrast mounted on an inverted Zeiss Axio Observer Z1 microscope (Marianas Imaging Workstation from 3i-Intelligent Imaging and Innovations Inc.), equipped with a CSU-X1 spinning-disk confocal head (Yokogawa Corporation of America) and four laser lines (405, 488, 561, and 640 nm) in an environment-controlled chamber (37 °C with controlled humidity and 5 % CO_2_ supply). Images were acquired using an iXon Ultra 888 EM-CCD camera (Andor Technology).

For this experiment, HeLa GFP-Tubulin mCherry-H2B cells were cultured in 35 mm glass-bottomed dishes (14 mm, No. 1.5, MatTek Corporation, Ashland, MA, USA) and treated with siRNA against Rab11a and KIF13A for 48 h before imaging. Cells were imaged at 2 min interval for 480 min with 1-μm z-plane slices, covering the entire mitotic spindle. All images display the maximum projections of z-stacks from representative data of at least three biological replicates per condition. Cell division duration was quantified by tracking the time spent from nuclear envelope breakdown to cytokinetic abscission. 

### Antibodies and staining reagents

A detailed list of antibodies and staining reagents is available in the Supplementary Information (Table S1).

### Multinucleation assay

To assess multinucleation, equal numbers of HeLa-wt cells-treated with either non-targeting shRNA control or two distinct high knockdown (KD) efficiency shRNAs, each separately targeting Rab11a and KIF13A-were seeded on collagen I-coated (at final 50 μg/ml concentration, Life Technologies Gibco, USA) No. 1 microscope cover glasses (VWR, USA). After 48 h of culture, cells were fixed with 4 % PFA (Thermo Fisher Scientific, USA). Fixed cells were then permeabilised with 0.2 % Triton X-100 (Thermo Fisher Scientific, USA, #A160046.AE) for 3 min at R.T. and stained with Alexa Fluor 568 phalloidin (Thermo Fisher Scientific, USA, #A12380) for 30 min at 37 °C and with 1 µg/ml DAPI (Thermo Fisher Scientific, USA, #D1306) for 5 min at R.T. The cover glasses were mounted on glass microscope slides using Prolong Diamond Antifade mountant (Invitrogen, USA, #P36965). Then, random fields on the coverslips were photographed using an inverted fluorescence Olympus IX73 microscope (Olympus Europe Holding GmbH) with a 20× lens. The number of multinucleated cells - including binucleated and poly-lobed cells - as well as micronuclei, was manually quantified. DAPI staining was used to label nuclear DNA, enabling accurate visualisation and enumeration of nuclei, while phalloidin staining of filamentous actin defined the cellular boundaries. Cells were classified as multinucleated when two or more distinct DAPI-positive nuclei were contained within a single phalloidin-demarcated cytoplasm. The rate of total multinucleated cells was then calculated in ten randomly chosen fields for each biological repeat. All cells were valued as one population. All data are derived from at least three independent experiments (biological repeats), each containing 5-15 technical replicates. Data are reported as the mean with the corresponding standard deviation. Asterisks (*) indicate statistically significant differences (p < 0.05). 

### Mitotic stage analysis

To quantify the percentage of telophase cells following shRNA treatment, HeLa-wt cells treated with a non-targeting shRNA control or various shRNA constructs were fixed and stained using an anti-acetylated α-tubulin antibody (Cell Signalling, USA, #D20G3, 1:200) and DAPI (Thermo Fisher Scientific, USA, #D1306), following standard protocols. Cells in telophase were identified based on the presence of a mitotic spindle and chromatin condensation. Cells were counted in 10 randomly selected fields in each independent experiment and expressed as a percentage of all cells. All data are derived from at least three independent experiments (biological replicates), each of which contains 5-15 technical replicates. Results are shown as mean ± standard deviation. Asterisks (*) indicate statistically significant differences (p < 0.05). 

### Colocalisation assay

A defined number of control, Rab11a-KD, and KIF13A-KD cells were seeded onto collagen I-coated glass coverslips. After 48 hours of incubation, the cells were fixed and stained with anti-Rab11a and anti-KIF13A antibodies (see Supplementary Table S1), together with DAPI (Thermo Fisher Scientific, USA, #D1306), to visualise and quantify the colocalisation of Rab11a and KIF13A at the ICB and the entry points (EPs - flanking regions of the ICB corresponding to the interface with the daughter cell) during cytokinesis. Colocalisation was quantified using the Pearson correlation coefficient (JACoP plugin in ImageJ, National Institutes of Health, USA), which measures the degree of overlap between the two fluorescence channels. 

In brief, cytokinetic cells that had fully reformed their cytoskeleton and no longer exhibited a cleavage furrow were selected to analyse the colocalisation of Rab11a and KIF13A at the ICB and EPs in control and Rab11a- or KIF13A-shRNA-treated cells. A single focal plane containing a cytokinetic cell with the entire ICB in focus, identified by the maximal fluorescence intensity, was manually selected from each image. Based on a visual inspection, the colour thresholds for Rab11a and KIF13a were jointly adjusted to avoid correlation variations due to inaccurate area size and to optimally capture signal at the ICB and the EPs while minimising background contribution. This indicates that the region of interest (ROI) is manually defined and consistently maintained across both channels within each image, ensuring an accurate representation of the proteins and confirming that it does not influence the correlation analysis, while permitting variation between different images. It also minimises potential bias in correlation analysis arising from human error in selecting cells that best represent the cytokinetic stage. To maintain the validity of this analysis, the same selection criteria were consistently applied throughout the experiment when assessing colocalization using the JACoP plugin. The resulting Pearson's correlation coefficient represents the codependency of variations in grey intensity across two-channel images using linear regression, yielding the correlation value “r”. If r = 1, the two channels are completely interdependent, if r = 0, no random colocalisation, and if r = -1, complete lack of colocalisation.

The data represent findings from at least three independent experiments (biological repeats), each comprising cells analysed across 5 distinct images (technical replicates). Values are presented as mean ± standard deviation, and asterisks (*) denote statistically significant differences (p < 0.05). 

### Lagging chromatin bridges

A defined number of control, Rab11a-KD, and KIF13A-KD cells were seeded onto collagen I-coated glass coverslips. Following 48 hours of incubation, cells were fixed and stained with anti-LAP2 and anti-tubulin antibodies (see Supplementary Table S1), along with DAPI (Thermo Fisher Scientific, USA, #D1306) to visualise and quantify stretched chromatin bridges between adjacent interphase cells. In each independent experiment, cells were assessed in at least five randomly selected fields (technical replicates), and the number of chromatin bridges was expressed as a percentage of the total cell population. Experiments were performed in triplicate (three biological replicates), where each replicate contains 5-15 technical replicates. Results are shown as mean ± standard deviation. Asterisks (*) indicate statistically significant differences (p < 0.05). 

### Statistical analysis

Unless otherwise indicated, all data are derived from at least three independent experiments (biological replicates), with the mean and standard deviation calculated. The data were processed using the Microsoft Office Excel data analysis tool pack (Microsoft Corporation, Redmond, WA, USA). A Student's *t*-test was used on all datasets to determine the significance level. Whenever three or more groups were compared, One-way ANOVA was used to perform statistical analysis. The level of significance was set as p < 0.05.

Data were collected from at least five randomly chosen image fields per coverslip for the multinucleation assay, mitotic stage analysis, and evaluation of lagging chromatin bridges. All the experiments were repeated at least three times (technical replicates). To quantify immunofluorescence, all specimens in the experiment were imaged with the same exposure settings, and the image data were analysed using ImageJ (National Institutes of Health, USA). 

## Results

### The regulatory functions of Rab11a and KIF13A during the regulation of cell division

**Figure 1:**
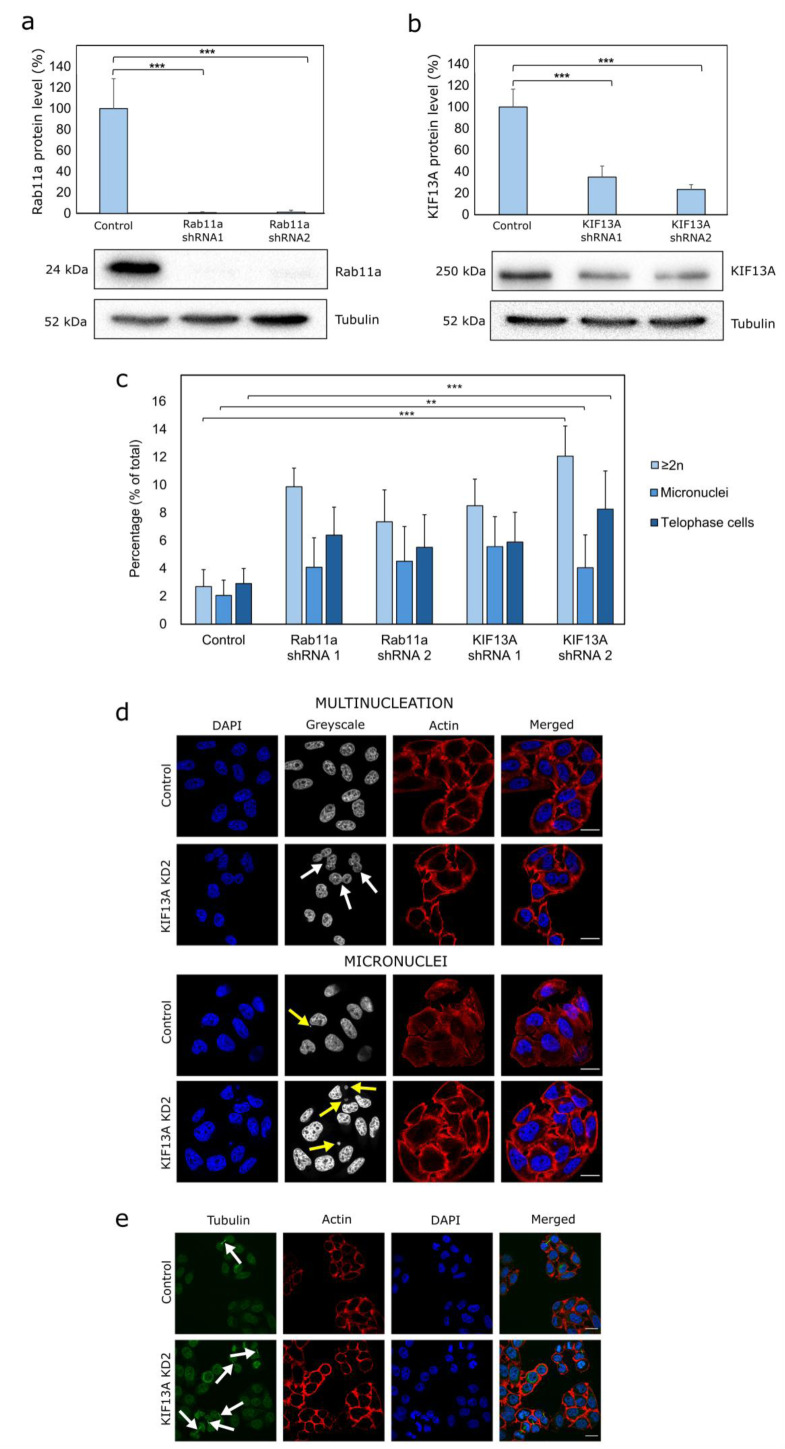
Rab11a or KIF13A knockdown via shRNA promotes multinucleation and elevates telophase arrest or delay. (a-b) Stable cell lines depleted of Rab11a or KIF13A using distinct short hairpin RNAs (shRNAs) were cultured to confluency. Lysates were then collected, and the remaining expression of Rab11a and KIF13A proteins was measured using Western blotting. Above each Western blot, relative protein levels are presented for cells in which Rab11a and KIF13A were silenced using shRNA. β-tubulin was used as a loading control, and the relative expression of the proteins was normalised to the level of intracellular tubulin. The data shown are the means and S.D. derived from at least three independent experiments. Asterisks (*) indicate statistically significant differences (p < 0.05). (c) Control and various shRNA-depleted HeLa-wt cells were fixed and stained with DAPI and phalloidin. The total number of binucleated and poly-lobed cells, micronucleated cells (counted separately), and cells delayed or arrested in telophase was quantified for each stable cell line population. Asterisks (*) indicate statistically significant differences (p < 0.05). The data shown are the means and S.D. derived from at least three independent experiments. (d) The images depict the HeLa-non-targeting shRNA control population alongside the HeLa-KIF13A-KD2 population, highlighting binucleated, poly-lobed, and micronuclei-containing cells (scale bar = 20 µm). White arrows indicate binucleated and poly-lobed cells, while yellow arrows mark micronucleated cells resulting from cytokinesis failure. A minimum of 30 images per group were taken using 20× magnification. A minimum of three independent experiments were conducted in total. (e) The images show the HeLa-non-targeting shRNA control population alongside the HeLa-KIF13A-KD2 population, with white arrows indicating cells delayed or arrested in telophase (scale bar = 20 µm). A minimum of 30 images per group were taken using 20× magnification. A minimum of three independent experiments were conducted in total.

To begin, we employed the short hairpin RNA (shRNA) approach to downregulate the Rab11 isoform Rab11a and KIF13A using distinct shRNAs. We used two shRNAs for the gene, each targeting distinct sequences to minimise the likelihood of off-target effects. According to the results, downregulation of Rab11a using shRNA1 and shRNA2 nearly eliminated the protein, reducing its levels by 99.3 % and 98.8 %, respectively (Figure 1a(Fig. 1)). This indicates that both shRNAs were similarly effective at downregulating Rab11a. However, KIF13A downregulation was less efficient, with shRNA1 and shRNA2 reducing protein levels by only 65.09 % and 76.66 %, respectively (Figure 1b(Fig. 1)), suggesting that shRNA2 achieved a stronger knockdown (KD). Accordingly, for the remainder of the study, we chose not to omit the less efficient KDs but instead included all knockdown conditions in the subsequent experiments. For full-length Western blots, please refer to Supplementary Information, Figure S1.

**Figure 2:**
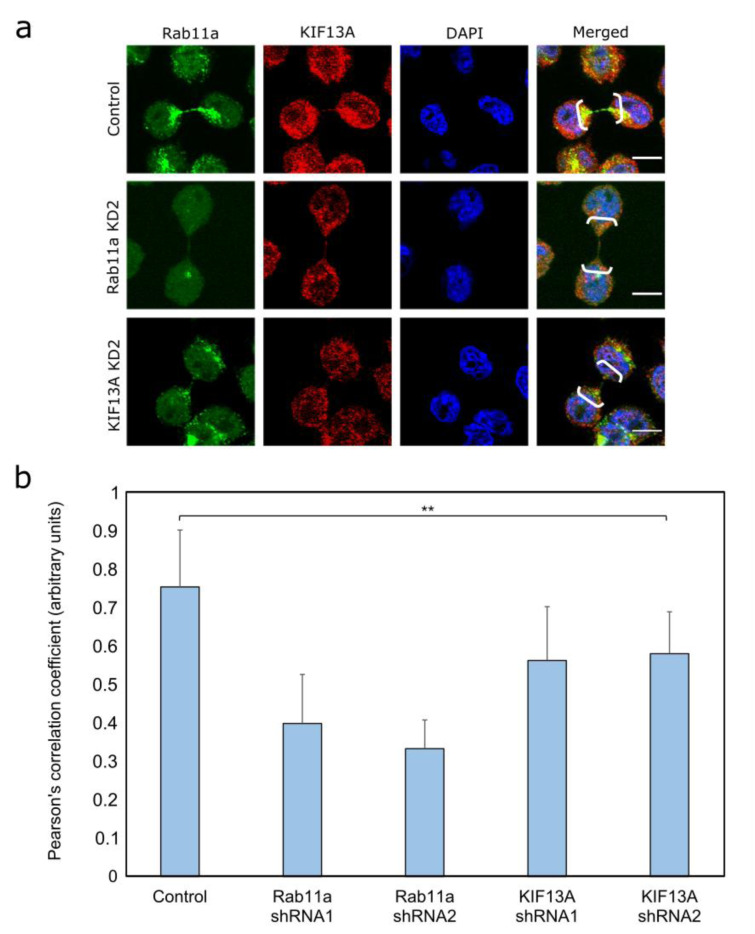
Rab11a and KIF13A colocalise at the EPs and ICB during cytokinesis. (a) Stable non-targeting control cells and cell lines depleted of Rab11a or KIF13A using distinct shRNAs were fixed and stained with DAPI and the protein-specific antibodies. The colocalisation pattern of Rab11a and KIF13A at the ICB and the EPs was analysed in cytokinesis (scale bar = 10 µm). The regions in white brackets highlight the cellular regions analysed for colocalisation using the Pearson Coefficient. (b) The graph presents colocalisation correlation analysis of Rab11a and KIF13A at the ICB and the EPs during cytokinesis across control and different shRNA-depleted stable cell lines. A minimum of 15 images per group were taken using 60× magnification. A minimum of three independent experiments (with 5 images each) were conducted in total. Asterisks (*) indicate statistically significant differences (p < 0.05).

These WB results are consistent with the localisation data, in which we used stable cell lines individually depleted of Rab11a and KIF13A to assess the localisation of each protein and its colocalisation patterns. Under normal conditions, Rab11a colocalise with KIF13A at the EPs and the entire body of the ICB during cytokinesis, where the ICB becomes long and narrow, facilitating the abscission (Figure 2a(Fig. 2)). In Rab11a-shRNA-treated cells, Rab11a does not appear at its normal cytokinesis sites (Figure 2a(Fig. 2)), which is consistent with the almost 100 % gene knockdown in WB (Figure 1a(Fig. 1)). However, when Rab11a is downregulated, KIF13A expression at these sites is markedly reduced, as evident from the immunofluorescence images (Figure 2a(Fig. 2)). Since shRNA treatment leaves approximately 25-35 % of KIF13A expression remaining, as shown by the WB results (Figure 1b(Fig. 1)), a substantial portion of the protein is still present at its usual cytokinetic location, as shown by immunofluorescence imaging (Figure 2a(Fig. 2)). The colocalisation data was further supported by Pearson's correlation coefficient measurements. Our results show that in Rab11a-shRNA1- and shRNA2-treated cells, the Pearson's correlation coefficients for Rab11a and KIF13A were 0.40 and 0.33 arbitrary units, respectively - nearly half of the 0.76 measured in control (Figure 2b(Fig. 2)). In contrast, in KIF13A-shRNA1- and shRNA2-treated cells, Pearson's correlation coefficients were 0.56 and 0.58 arbitrary units, only about 30 % lower than the control value of 0.76 (Figure 2b(Fig. 2)). Overall, the colocalisation correlation findings aligned well with both the localisation data (Figure 2a-b(Fig. 2)) and earlier WB results (Figure 1a-b(Fig. 1)).

### Downregulation of Rab11a or KIF13A induces multinucleation in dividing cells

After confirming the downregulation and completing initial expression analysis of Rab11a and KIF13A in KD cells, we next examined how this reduction affected cytokinesis using various phenotypic analyses. We used the multinucleation assay as the initial approach to evaluate cells for defects in cytokinesis. Multinucleation is a direct consequence of defective cytokinesis due to abscission failure or cleavage-furrow regression (D'Avino et al., 2005[10]), meaning that nuclear division proceeds without subsequent cytoplasmic separation. Under normal conditions, cytokinesis divides the parental cell's cytoplasm to produce two distinct daughter cells (Green et al., 2012[17]; Lens and Medema, 2019[27]). However, when misregulated, cytokinesis failure can lead to multinucleation, which in cancer cells has been linked to the development of aneuploidy (Wilmerding et al., 2026[48])-a feature associated with increased tumour aggressiveness (Li et al., 2000[29]). In this assay, for nuclear visualisation, cells were stained with DAPI, along with phalloidin, which allowed for distinguishing nuclei as well as separating clumped cells. According to the results, compared with the control, in which 2.69 % of cells were multinucleated, Rab11a-shRNA1- and Rab11a-shRNA2-treated cells exhibited 9.87 % and 7.35 % multinucleated cells, respectively (Figure 1c-d(Fig. 1)). The results were comparable to those observed for KIF13A KDs, where 8.51 % and 12.07 % of the cells were in the ≥ 2n state, following treatment with KIF13A-shRNA1 and KIF13A-shRNA2, respectively (Figure 1c-d(Fig. 1)). These statistically significant results suggest that cells with reduced expression of genes involved in membrane trafficking during cytokinesis are partially unable to complete division, resulting in an increased number of multinucleated cells.

### Cells lacking functional Rab11a or KIF13A lead to an increased formation of micronuclei in cells

We next examined genomic instability, which may result from disrupted cell division and can be identified by the presence of micronuclei. It has been demonstrated that genomic instability, resulting from impaired DNA damage responses or chromosomal missegregation during mitotic cell division, can lead to the formation of aberrant extranuclear DNA-containing structures known as micronuclei (Adams et al., 2024[1]). Alternatively, these structures are formed from chromosome fragments that result from mitotic segregation errors (Fenech et al., 2011[14]; Klaasen et al., 2022[26]) or unrepaired DNA breaks (Crasta et al., 2012[9]; Ly et al., 2017[30]). These misregulated processes often lead to the formation of mitotic chromosome bridges, which subsequently give rise to micronuclei. Micronuclei generated after mitosis from lagging chromatids or chromatin bridges between anaphase chromosomes can persist in cells until the completion of the cell cycle (Utani et al., 2010[46]). Thus, based on the points outlined above, the formation of chromatin bridges in cells depleted of Rab11a or KIF13A will also be examined later in this study. However, for now, to assess genomic instability using micronuclei as a physical marker, we stained the cells with DAPI and phalloidin. This assay had the added benefit that the same samples with cells, stained for the multinucleation assay, could also be used to evaluate micronuclei formation (Figure 1d(Fig. 1)). According to the data, treatment with Rab11a-shRNA1, Rab11a-shRNA2, KIF13A-shRNA1, and KIF13A-shRNA2 resulted in a statistically significant increase in micronuclei formation - 4.08 %, 4.51 %, 5.58 %, and 4.05 %, respectively - compared to the control at 2.06 % (Figure 1(Fig. 1)). These findings indicate that protein trafficking defects to the ICB and the MB, which affect the process of cell division, also influence genomic stability, suggesting that cytokinesis is not the only phase affected. Based on the results, it is plausible that chromosome segregation errors during anaphase also contributed to these phenotypic differences, and additional investigation of lagging chromatin bridges could help validate this.

### Reducing Rab11a and KIF13A expression results in delayed progression through telophase

Having observed noticeable changes in multinucleation and micronuclei formation following the downregulation of Rab11a and KIF13A, which provided preliminary evidence of their involvement in cell division, we next investigated their potential effects on telophase. This phase bridges anaphase and cytokinesis, and is the phase where chromosomes unwind to form chromatin. Meanwhile, mitotic spindle microtubules extend and then compact into the ICB, with the MB positioned in the middle (Tipton and Gorbsky, 2022[45]). Theoretically, disruption of the molecular mechanisms regulating telophase could either block the transition from anaphase to ICB formation in telophase or hinder progression from telophase to cytokinesis, resulting in a higher number of cells being delayed or arrested in telophase. Surprisingly, Rab11a and KIF13A KDs caused a significant accumulation of cells in telophase, with 6.39 %, 5.51 %, 5.89 %, and 8.26 % of cells delayed or arrested following treatment with Rab11a-shRNA1, Rab11a-shRNA2, KIF13A-shRNA1, and KIF13A-shRNA2, respectively (Figure 1c, e(Fig. 1)). These values were significantly higher than in the control, which showed 2.91 % (Figure 1c, e(Fig. 1)). Overall, the combination of functional assays provides evidence that Rab11a and KIF13A are equally important for regulating cell division, with their most prominent role in cytokinesis, as demonstrated by the most significant increase in multinucleation upon the depletion of the latter proteins (Figure 1c(Fig. 1)).

### Depletion of Rab11a or KIF13A results in the formation of chromatin bridges during mitosis

**Figure 3:**
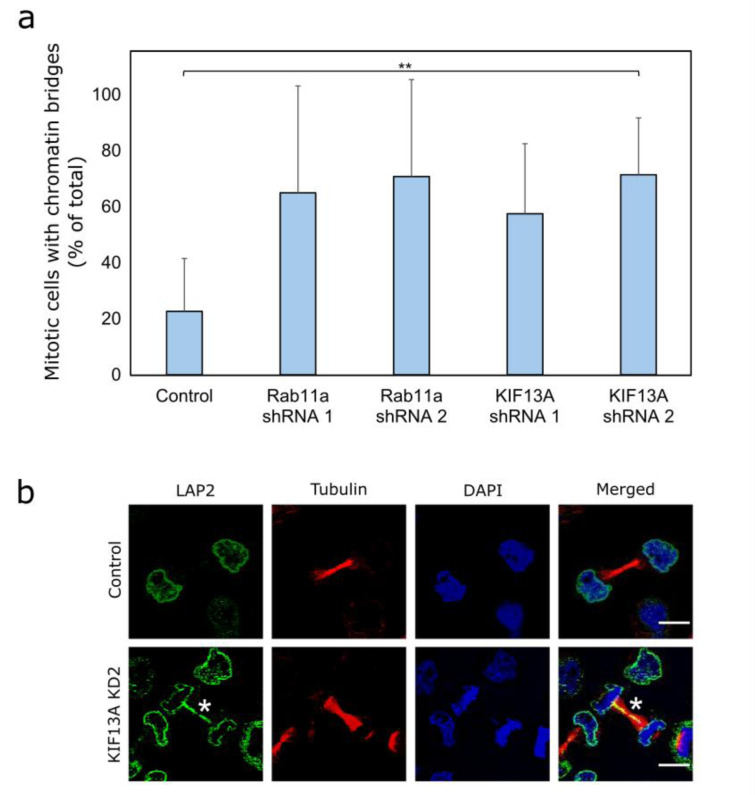
Downregulating Rab11a or KIF13A via shRNA induces the formation of chromatin bridges associated with lagging chromatin dissociation during mitosis. (a) Stable non-targeting control cells and cell lines depleted of Rab11a or KIF13A using distinct shRNAs were fixed and stained with DAPI, anti-acetylated-α-tubulin, and a LAP2-specific antibody to visualise chromatin-associated regions. The number of cells showing lagging chromatin at their ICB was determined by manual counting. Asterisks (*) indicate statistically significant differences (p < 0.05). The data shown are the means and S.D. derived from at least three independent experiments. (b) A chromatin bridge is observed in the KIF13A-shRNA 2 stable knockdown cell line, but not in the non-targeting shRNA control, during telophase (scale bar = 10 µm). A white asterisk (*) indicates chromatin bridge formed during mitosis.

The presence of micronuclei is considered a hallmark of chromosome instability (Soto et al., 2018[40]). Given the increase in micronuclei formation observed following Rab11a or KIF13A KDs, it is plausible that genomic instability was triggered in dividing HeLa cells. As noted earlier, micronuclei, which are abnormal extranuclear structures containing DNA, form as a result of mitotic segregation errors (Fenech et al., 2011[14]; Klaasen et al., 2022[26]) or persistent DNA breaks (Crasta et al., 2012[9]; Ly et al., 2017[30]). These unstable DNA deposits could be derived from the chromatin bridges formed between separating anaphase chromosomes (Utani et al., 2010[46]). To assess whether chromatin bridges indeed gave rise to micronuclei, we subsequently labelled the cells with LAP2-specific antibody, along with DAPI and acetylated-α-tubulin and examined the formation of chromatin bridges within the mitotic population. Acetylated-α-tubulin is preferentially used to label ICBs during cytokinesis because it selectively marks a population of long-lived, stable microtubules that are highly enriched within the MB. In contrast, β-tubulin-positive microtubules are widely distributed throughout the cells, producing a high background and making the narrow ICB harder to distinguish. Acetylated-α-tubulin therefore provides high signal-to-noise labelling of the MB microtubule bundle, allowing clear visualisation of ICBs even at late stages of cytokinesis. According to our results, chromatin bridge formation rose markedly relative to the 22.67 % observed in control cells (Figure 3a-b(Fig. 3)), attaining 65.00 %, 70.77 %, 57.50 % and 71.43 % in Rab11a-shRNA1-, Rab11a-shRNA2-, KIF13A-shRNA1- and KIF13A-shRNA2-treated cells, respectively (Figure 3a-b(Fig. 3)). These results align well with micronuclei formation assay results and help explain the observed increase in micronuclei formation in cells depleted of genes involved in regulating cell division.

### Reducing Rab11a or KIF13A expression results in an extended cytokinesis and longer overall cell division

Given that all these phenotypic alterations were clearly expressed in cells with reduced Rab11a or KIF13A expression, we next employed live-cell imaging to capture actual divisions and measure the time required for cells to transition from one phase to another, recording the overall duration of mitotic M-phase. To better preserve natural cellular conditions and avoid disrupting the overall division process, we chose to downregulate Rab11a and KIF13A using siRNAs rather than shRNA. Indeed, siRNA short-term gene suppression minimises compensatory mechanisms that can arise with long-term shRNA expression (Boudreau et al., 2009[3]). Moreover, shRNA utilises viral delivery, which often results in heterogeneous expression, depending on the integration site and copy number, thereby creating variability between cells. In our study, this would be incompatible with single-cell live-cell imaging (Cahill et al., 2007[5]). Also, we used two siRNAs targeting distinct sequences for the Rab11a and KIF13A genes, respectively, to minimise the likelihood of off-target effects. Before the actual live-cell imaging, we also evaluated the effectiveness of downregulation using different siRNA concentrations of 25 nM and 50 nM, along with incubation periods of 48 and 72 hours. Briefly, according to the results, the 25 nM siRNA concentration applied for 48 h was the most optimal condition for both Rab11a-siRNA1- and KIF13A-siRNA1-treated cells (Figure S2a-b). At the lowest concentration, it produced the most efficient KD in the shortest time. Although KIF13A expression decreased following Rab11a downregulation, and reciprocally Rab11a levels declined upon KIF13A suppression, these changes did not reach statistical significance (Figure S2a-b). For full-length Western blots, please refer to Figure S3.

**Figure 4:**
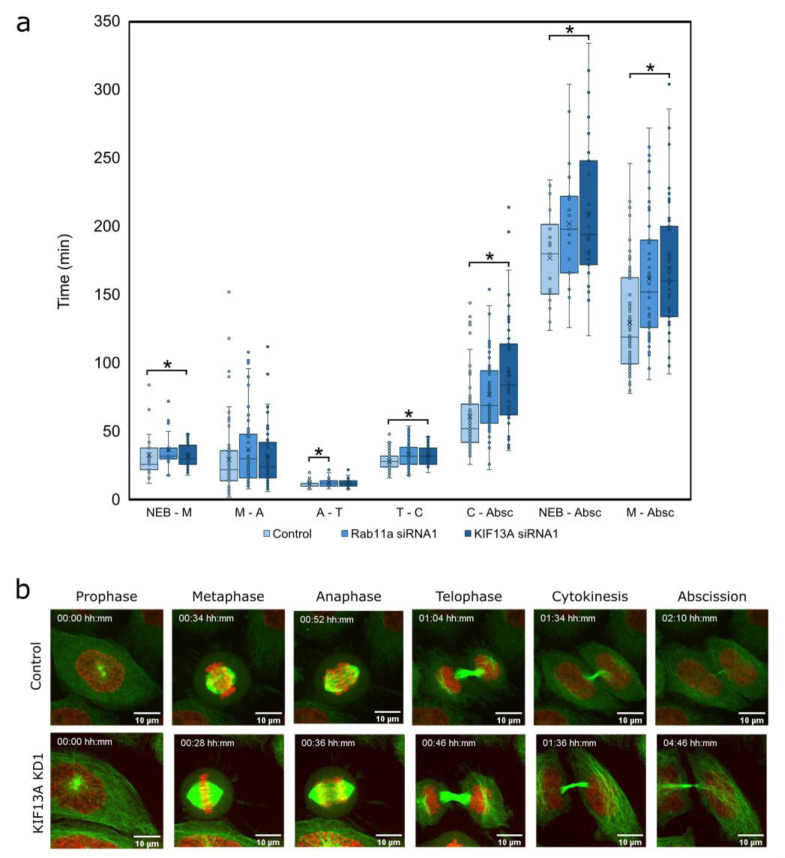
Rab11a and KIF13A knockdown result in an extended cell division time. Individual knockdown of Rab11a and KIF13A results in prolonged cell division time, with the most notable difference occurring during the transition from cytokinesis to abscission in HeLa cells expressing GFP-tubulin-mCherry-H2B. The graph in panel a summarises the results of cell division. Asterisks (*) denote statistically significant differences (p < 0.05). Data are presented as means ± S.D. from at least three independent experiments. Panel b shows the stages of mitotic M-phase and the corresponding time intervals for progression between each phase. Screenshots from time-lapse movies show the expected division timing in control cells and prolonged division time in KIF13A-depleted cells treated with siRNA1. Scale bar indicates 10 µm.

For the reasons above, the live-cell signalling was conducted using cells depleted with Rab11a-siRNA1 or KIF13A-siRNA1 only. Cell division duration was quantified by tracking the time spent from nuclear envelope breakdown to cytokinetic abscission. Based on the results, the time required to progress from nuclear envelope breakdown in prophase to metaphase, from metaphase to anaphase, from anaphase to telophase, and from telophase to cytokinesis did not differ substantially between control cells and those treated with Rab11a-siRNA1 or KIF13A-siRNA1 (Figure 4a(Fig. 4)). As expected, the most pronounced and statistically significant difference occurred during the transition from cytokinesis to abscission: control cells required an average of 60.85 min, compared with 77.28 and 92.38 min in Rab11a-siRNA1- and KIF13A-siRNA1-treated cells, respectively (Figure 4a(Fig. 4)). These differences in the cytokinesis-to-abscission transition are clearly illustrated in the live-cell imaging screenshots (Figure 4b(Fig. 4)), as well as time-lapse movies (Supplementary Movies 1 and 2). In summary, the total time from nuclear envelope breakdown in prophase to final abscission averaged 176.89, 201.90, and 208.13 min in control, Rab11a-siRNA1-treated, and KIF13A-siRNA1-treated cells, respectively, with statistically significant differences between the control and knockdown conditions (Figure 4a(Fig. 4)). When assessing the total duration of the mitotic M-phase, it also increased significantly - from 129.68 min in control cells to 161.57 and 168.98 min in Rab11a-siRNA1- and KIF13A-siRNA1-depleted cells, respectively (Figure 4a(Fig. 4)). This assay enabled us to examine phenotypic differences resulting from gene depletion, focusing on a scientific question that aims to clarify the role of Rab11a and KIF13A in regulating cell division. The results align with the data from the previous functional assays, adding value to the reliability of the findings.

### The interacting proteins help to uncover the molecular machinery underlying the KIF13A-Rab11a axis 

**Figure 5:**
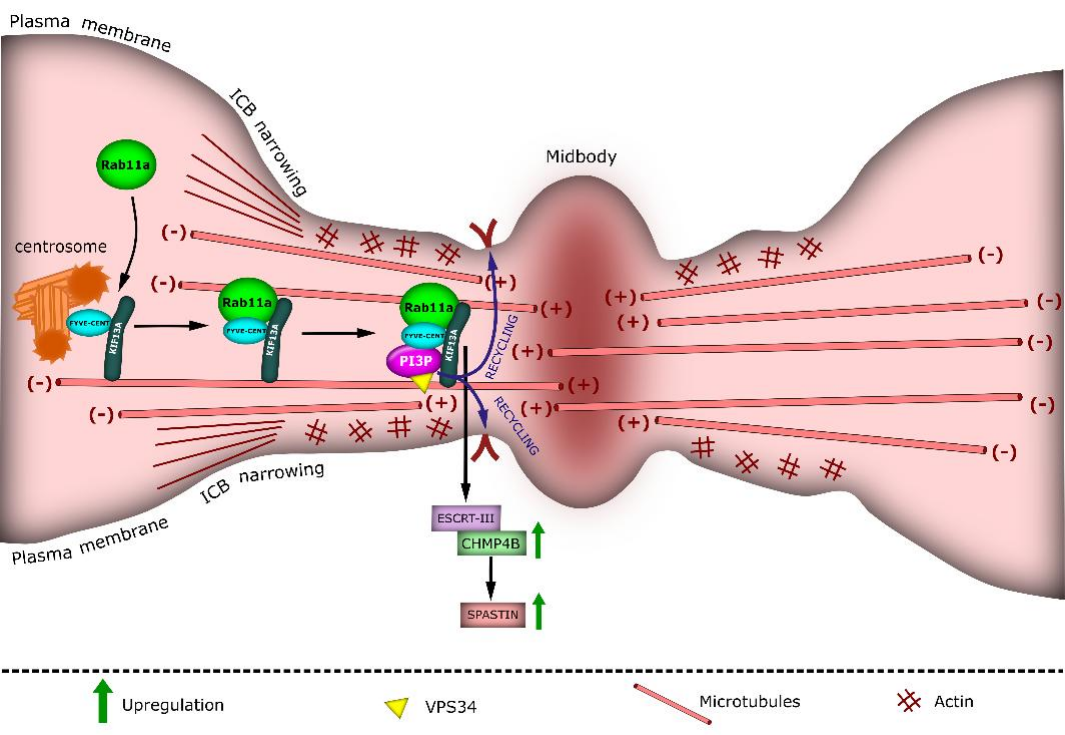
A model representing the proposed KIF13A-Rab11a axis-mediated transport to the abscission site at the ICB. In our proposed model, the interaction between centrosome-associated FYVE-CENT and the microtubule plus-end-directed motor KIF13A drives FYVE-CENT transport toward the MB. Similar to this, Rab11a-positive endosomes bind to KIF13A, facilitating their coordinated movement along the same route. Meanwhile, the Vps34 activity generates localised PI3P on endosomes within the ICB, providing anchoring sites for FYVE-CENT. The recruitment of the KIF13A-FYVE-CENT complex is therefore likely to regulate the recycling of PI3P-enriched endosomes that are essential for successful abscission. Furthermore, the delivery of Rab11a to the MB, along with other KIF13A-associated cargo, narrows the ICB sufficiently for activated CHMP4B, a component of the ESCRT-III complex, to initiate a signalling cascade that also facilitates actin clearance and, through spastin activation, drives microtubule severing within the ICB.

According to the literature, a microtubule plus-end directed motor protein, KIF13A, mediates the movement of the FYVE-CENT-TTC19 complex towards the MB, where the activity of Vps34 leads to local production of PI3P endosomes within the ICB (Thoresen et al., 2014[44]), which then serve as a binding site for FYVE-CENT via its FYVE binding domain (Montagnac and Chavrier, 2010[31]; Sagona et al., 2010[38]). This recruitment subsequently regulates the recycling of PI3P endosomes and activates the ESCRT-III complex component CHMP4B, which is required for abscission (Sagona et al., 2010[38]; Montagnac and Chavrier, 2010[31]; Carlton et al., 2012[6]). Building on this already published evidence supporting our proposed KIF13A-Rab11a regulatory machinery, we next sought to examine the presence and spatial distribution of putative downstream effectors of the KIF13A-Rab11a axis during cytokinesis. To this end, we labelled control, Rab11a-siRNA1- and KIF13A-siRNA1-treated cells with antibodies against CHMP4B, Aurora B and PI3P. These markers were selected intentionally: the absence of activated CHMP4B would indicate disruption of the KIF13A-driven signalling cascade, whereas the presence of Aurora B would signify activation of the abscission checkpoint and concomitant downregulation of Vps34 activity and thus subsequent dysregulation of PI3P production, collectively indicating upstream defects in the signalling pathway (Figure 5(Fig. 5)). According to our results, CHPM4B appeared to accumulate at both the EPs and the ICB in control cells during cytokinesis (Figure S4a). In Rab11a-depleted cells, however, CHMP4B was absent from both the EPs and the ICB (Figure S4b), whereas in KIF13A-depleted cells, CHMP4B was specifically missing from the ICB (Figure S4c). This altered localisation of CHMP4B, relative to the control, may indicate a disruption of the upstream signalling cascade required for CHMP4B activation. At the same time, in control cells undergoing cytokinesis - characterised by long and narrow ICBs - Aurora B was detected at the tips of the ICB on both sides of the MB (Figure S4a). This localisation pattern changed upon Rab11a or KIF13A depletion: Aurora B was detected to a greater extent across the whole cell body, including the ICB, reflecting an evident upregulation (Figure S4b-c). This elevated expression of the abscission-delay regulator Aurora B may also indicate disruptions in the upstream signalling pathway during cytokinesis in Rab11a- or KIF13A-depleted cells. This observed upregulation of Aurora B aligns with the reduced PI3P levels detected in Rab11a- or KIF13A-depleted cells (Figure S4a-c), as the activated Aurora B kinase can interfere with the proper localisation of Vps34 at the MB, which precedes PI3P generation (Brill et al., 2011[4]; Thoresen et al., 2014[44]). Integrating our data with earlier evidence (Sagona et al., 2010[38]), we propose a model in which the KIF13A-Rab11a signalling cascade drives cytokinesis and ultimately promotes the final abscission of the ICB that connects the newly formed daughter cells (Figure 5(Fig. 5)). For the final abscission to occur, the subsequent recruitment of the ESCRT-III-related protein IST1 enables the direct binding of the AAA ATPase spastin, which mediates microtubule severing (Figure 5(Fig. 5)). Loss of spastin function consequently disrupts spindle disassembly (Vietri et al., 2015[47]).

## Discussion

Our data show that Rab11a and KIF13A are spatially and functionally coupled at the ICB. That perturbation of either component produces defects in late mitosis, including loss of ICB localisation, increased multinucleation and micronuclei, elevated rates of chromatin bridges, and a prolongation of the cytokinesis-to-abscission time. These results are consistent with prior models, in which recycling endosome trafficking and plus-end-directed microtubule motors jointly orchestrate membrane delivery and ESCRT activation at the MB (Montagnac and Chavrier 2010[31]; Sagona et al., 2010[38]; Delevoye et al., 2014[12]).

### Consistent with prior work on Rab11-mediated late-stage endosomal delivery

Several groups have demonstrated the requirement for Rab11-dependent recycling endosomes in abscission: Rab11 and its effectors (FIP3/FIP4) concentrate at the cleavage furrow and MB and are essential for successful abscission (Collins et al., 2012[8]; Simon and Prekeris, 2008[39]; Fielding et al., 2005[15]). Our observation that near-complete Rab11a depletion abolishes the Rab11a signal at cytokinetic sites and strongly reduces Rab11a-KIF13A colocalisation directly matches these earlier studies, which concluded that Rab11 organises endosomal identity and recruits effectors needed for membrane delivery during late cytokinesis (Wilson et al., 2005[49]).

### KIF13A couples PI3P/FYVE-CENT signalling at the midbody

A study by Sagona et al. showed that PI3P localises to the MB and that KIF13A mediates the recruitment of the FYVE-CENT/TTC19 complex to the MB, enabling CHMP4B/ESCRT-III activity required for abscission (Sagona et al., 2010[38]). Our finding that KIF13A depletion reduces CHMP4B accumulation at the ICB - and that KIF13A KD produces pronounced abscission delays and multinucleation - fits this mechanistic framework and supports the conclusion that KIF13A-driven transport is critical for establishing a PI3P-competent environment at the MB.

### Rab11a-KIF13A coupling in endosomal morphogenesis

Work by Delevoye et al. shows that KIF13A cooperates with Rab11 to generate recycling endosome tubules and to maintain RE morphogenesis, and that KIF13A can interact preferentially with GTP-bound Rab11a in interphase settings (Delevoye et al., 2014[12]). Our colocalisation data, especially the loss of KIF13A signal at cytokinetic sites after Rab11a depletion, and the more modest reciprocal effect, support a model in which Rab11a marks cargo endosomes and helps recruit KIF13A-containing transport complexes for directed delivery to the ICB. The asymmetry in dependency (more substantial loss of KIF13A localisation after Rab11a KD than vice versa) mirrors the concept that Rab GTPases confer membrane identity and thereby recruit motors and adaptors. 

### Impact on abscission timing, ESCRT recruitment, and checkpoint activation

We observed that both Rab11a and KIF13A depletion selectively prolong the cytokinesis-abscission interval (control - 60.85 min, Rab11a KD - 77.28 min, and KIF13A KD - 92.38 min). This phenotype parallels prior observations that impairing ESCRT-III recruitment or PI3P production delays abscission and that the abscission checkpoint (mediated by Aurora B) is engaged under such conditions (Steigemann et al., 2009[41]; Sagona et al., 2010[38]). The elevated Aurora B signal and decreased PI3P levels in our KDs are therefore consistent with checkpoint activation secondary to defective trafficking. When endosomal delivery or PI3P enrichment is compromised, Aurora B activity persists, delaying abscission and preventing catastrophic chromosome breakage. These results align with classical work describing the Aurora B-mediated abscission checkpoint and with studies linking Vps34/PI3P, FYVE-domain proteins and ESCRT recruitment to proper abscission (Elia et al., 2011[13]).

### Trafficking defects drive chromatin bridges and genome instability

The dramatic increase in LAP2-positive chromatin bridges (from 22.67 % in the control to 57.50-71.43 % in KDs) and the concomitant rise in micronuclei are notable because they connect membrane-trafficking defects at the ICB with segregation problems that lead to genome instability. This sequence - traffic defect, delayed abscission, persistent chromatin bridges, and micronuclei - has been previously proposed in studies demonstrating that delayed abscission promotes bridge persistence and subsequent nuclear abnormalities (Steigemann et al., 2009[41]; Pampalona et al., 2012[35]). Our data provide experimental support that defective Rab11a-KIF13A trafficking is sufficient to trigger this cascade in HeLa cells, reinforcing the pathological relevance of this pathway in the context of cancer, where both trafficking and mitotic fidelity are perturbed (Sadler et al., 2018[37]; Joseph et al., 2023[25]).

### Scientific insights, outstanding questions and future directions

By integrating our observations with the established KIF13A-FYVE-CENT-PI3P-ESCRT axis, we propose that Rab11a marks recycling endosomes that KIF13A must transport into the MB region to nucleate a PI3P-rich microdomain, recruit FYVE-domain effectors and enable ESCRT-III polymerisation and spastin-mediated microtubule severing (Figure 5(Fig. 5)). Disruption at any point in this chain produces delayed abscission and downstream genome instability. Given the prevalence of Rab and kinesin dysregulation in cancers (Xu et al., 2024[50]), the Rab11a-KIF13A module may represent a mechanistic link between altered membrane trafficking and aneuploidy in tumours. 

Although our results support the existing literature, several key scientific insights of our work are worth highlighting. First, KIF13A KD resulted in larger delays in abscission compared to Rab11a KD. Several possible reasons might be proposed for this difference: 1) The shRNA KD did not eliminate KIF13A (only a 65-76 % reduction), which could complicate how the phenotype compares to Rab11a KD. Partial depletion might still disrupt multiple pathways; 2) When Rab11a is depleted, other Rab11 family members (Rab11b or Rab25) or alternative regulatory factors may partially take over its role, reducing the severity of the phenotype. These kinds of compensatory mechanisms are common in trafficking pathways; 3) KIF13A may transport additional cargoes that do not rely on Rab11a, such as FYVE-CENT/TTC19 complexes, which are themselves required for abscission. Losing KIF13A would therefore disrupt multiple essential inputs into abscission, not just Rab11a-dependent trafficking. To elaborate on the topic, prior studies have noted isoform redundancy and the existence of multiple motors capable of supporting endosomal transport (Delevoye et al., 2014[12]; Thankachan and Setty 2022[43]; Gibieža et al., 2024[16]). Therefore, partial compensation is plausible and could explain why Rab11a depletion phenotypes are somewhat milder in some readouts. 

In summary, our data corroborate and extend published models by showing that Rab11a and KIF13A are spatially and functionally interdependent at the ICB, and that their coordinated action is required to generate a PI3P/ESCRT-competent environment that drives timely abscission. When this axis is disrupted, cells exhibit classical hallmarks of cytokinesis failure and genomic instability, underscoring the physiological and pathological significance of Rab11a-KIF13A-mediated trafficking in cell division. Future biochemical pull-downs and rescue experiments with separation-of-function mutants (utilising KIF13A CRISPR cells or locking Rab11a in GTP or GDP conformation) would help resolve whether the primary defect lies in motor recruitment, motor activation, or cargo maturation.

## Conclusions

Our results identify Rab11a and KIF13A as essential and functionally linked regulators of late mitosis, with a critical role in cytokinesis and abscission. Depletion of either protein disrupts their localisation at the ICB, delays abscission, and leads to increased multinucleation, chromatin bridges, and micronuclei formation, indicating defective cell division and compromised genomic stability. Live-cell imaging confirms that loss of Rab11a or KIF13A specifically prolongs the cytokinesis-to-abscission transition, extending overall mitotic duration. At the molecular level, mislocalisation of CHMP4B, reduced PI3P, and elevated Aurora B signalling support a model in which the Rab11a-KIF13A axis coordinates endosomal trafficking and ESCRT-III activation to ensure timely and faithful abscission. Together, these findings propose the Rab11a-KIF13A pathway as an additional regulatory mechanism that compensates existing pathways by linking membrane trafficking to the successful completion of cell division.

## Declaration

### Artificial Intelligence (AI) - assisted technology

We confirm that we have not used artificial intelligence tools in the preparation of the manuscript or in any part of the production process.

### Conflict of interest

The authors declare that they have no conflict of interest.

### Funding

This project was funded by the European Union (project No [S-PD-24-71]) under the agreement with the Research Council of Lithuania (LMTLT).

### Author's contributions

Conceptualisation, P.G.; Methodology, P.G.; Live-cell imaging conditions, S.R.C.; Validation, P.G.; Formal analysis, P.G.; Investigation, P.G.; Data curation, P.G.; Writing - original draft preparation, P.G.; Writing - review and editing, P.G., S.R.C. and V.P.; Visualisation, V.P.; Supervision, P.G. and V.P.; Project administration, P.G.; Funding acquisition, P.G. and V.P. All authors have read and agreed to the published version of the manuscript.

### Availability of data and materials

The datasets used and/or analysed during the current study are available from the corresponding author on reasonable request.

## Supplementary Material

Supplementary information

Movie S1

Movie S2
