# Supplementary material for: The KIF13A-Rab11a axis: a key regulator of vesicle trafficking in cytokinesis
Source: EXCLI J. 2026 Jun 19;25:824–45. doi: 10.17179/excli2026-9394 (PMC13369889; doi:10.17179/excli2026-9394)
Supplement: Supplementary information [file EXCLI-25-824-s-001.pdf]

EXCLI Journal 2026;25:824-845 – ISSN 1611-2156
Received: March 05, 2026, accepted: May 22, 2026, published: June 19, 2026

Supplementary information to:

Original article:

THE KIF13A-RAB11A AXIS: A KEY REGULATOR OF VESICLE TRAFFICKING IN CYTOKINESIS

Paulius Gibieža[1,2], Sergi Rodriguez-Calado[3], Vilma Petrikaitė[1,2]

[1] Institute of Biotechnology, Life Sciences Center, Vilnius University, Saulėtekio Ave. 7, LT-10257, Vilnius, Lithuania
[2] Laboratory of Drug Targets Histopathology, Institute of Cardiology, Lithuanian University of Health Sciences, Sukilėlių Ave. 13, LT-50162, Kaunas, Lithuania
[3] Cell Division and Cytoskeleton, Danish Cancer Institute, Strandboulevarden 49, 2100, Copenhagen, Denmark

* **Corresponding author:** Paulius Gibieža, Institute of Biotechnology, Life Sciences Center, Vilnius University, Saulėtekio Ave. 7, Vilnius, LT-10257, Lithuania. Tel.: +37062910490; E-mail: paulius.gibieza@lsmu.lt

https://dx.doi.org/10.17179/excli2026-9394

This is an Open Access article distributed under the terms of the Creative Commons Attribution License (https://creativecommons.org/licenses/by/4.0/).

EXCLI Journal 2026;25:824-845 – ISSN 1611-2156
Received: March 05, 2026, accepted: May 22, 2026, published: June 19, 2026

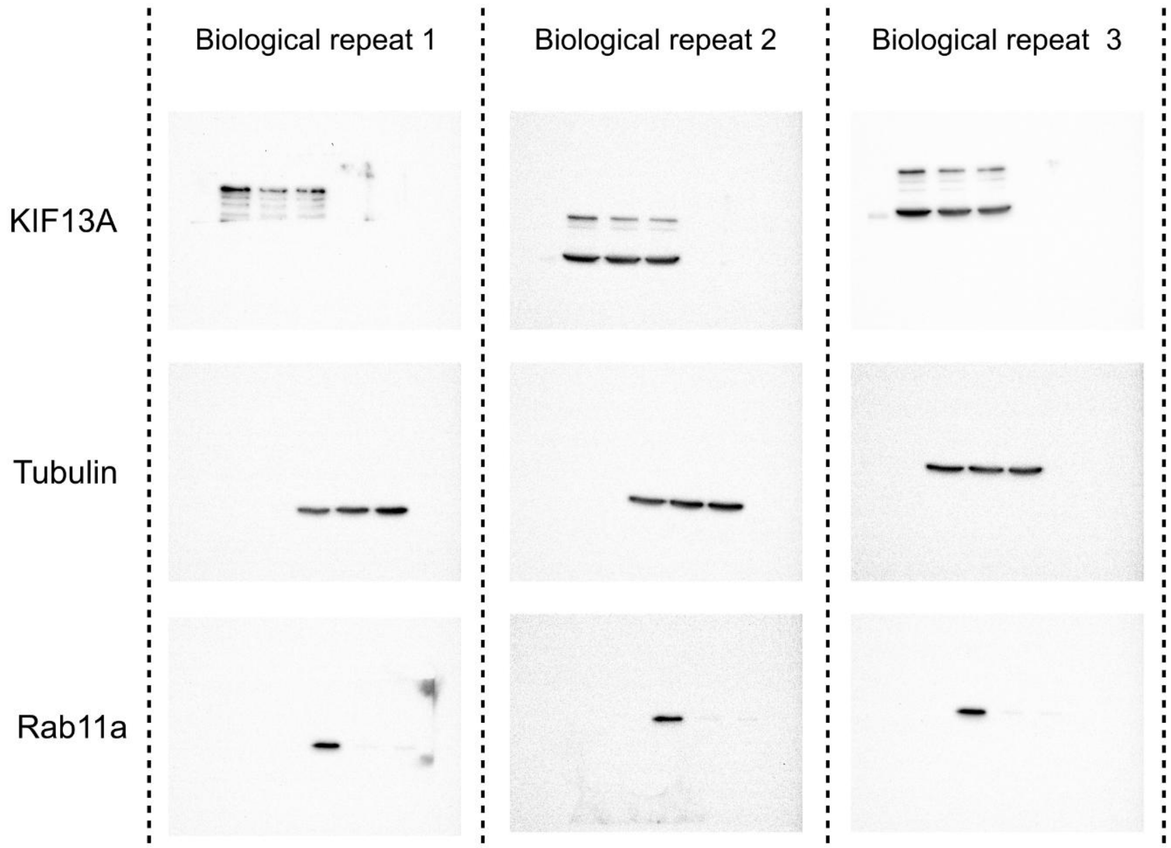

Supplementary Figure 1: Western blot images representing Rab11a and KIF13A knockdown by different shRNAs. The figure presents full-length Western blot images of KIF13A (at 250 kDa), beta tubulin loading control (at 55 kDa), and Rab11a (at 23 kDa) in control and Rab11a- or KIF13A-shRNA-depleted cells across three biological replicates.

Received: March 05, 2026, accepted: May 22, 2026, published: June 19, 2026

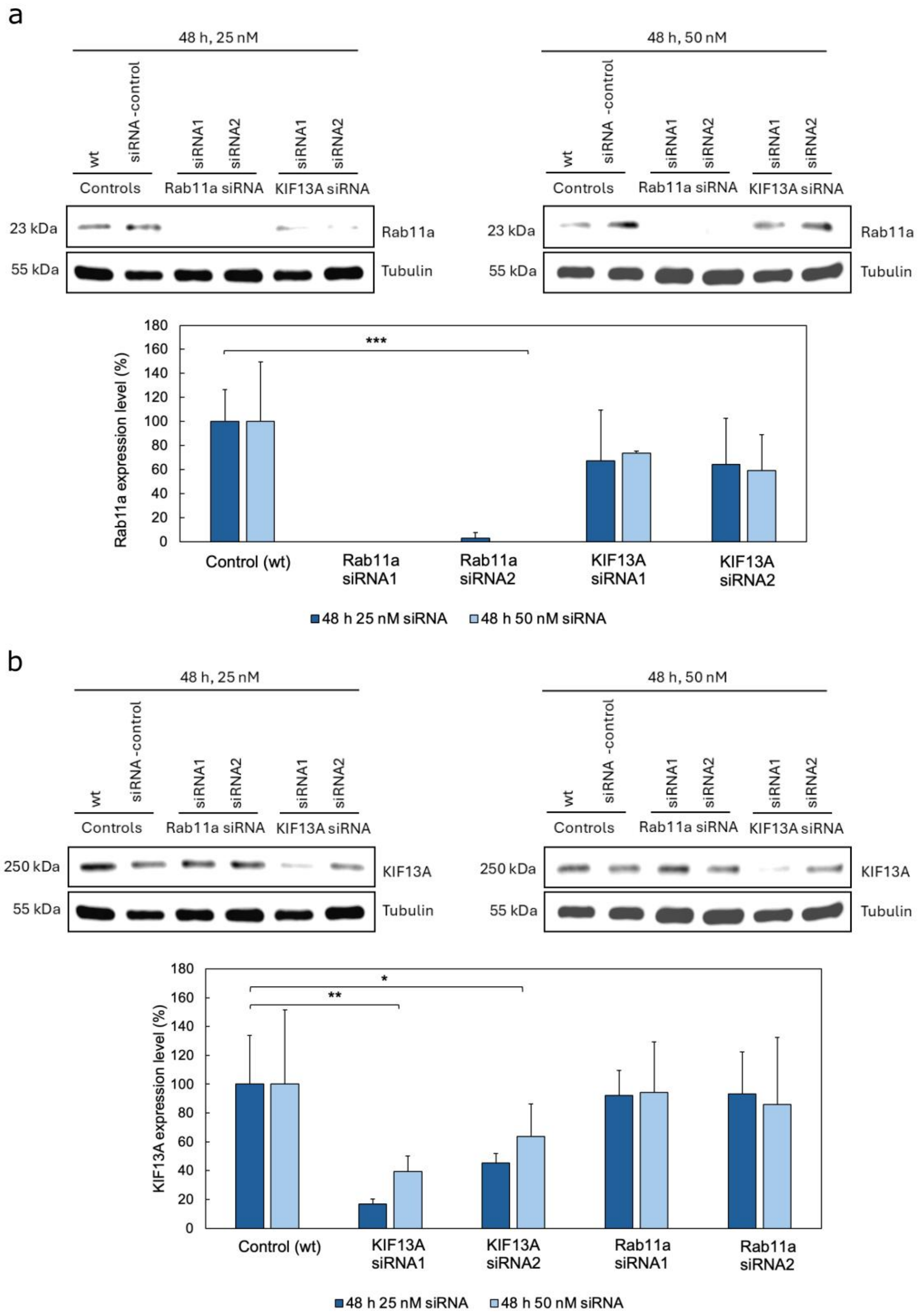

Supplementary Figure 2: Rab11a and KIF13A knockdown by siRNA under different experimental conditions. (a-b) Before live-cell imaging, the efficiency of Rab11a or KIF13A gene silencing was assessed using different siRNAs targeting distinct regions of the same gene, applied at concentrations of 25 nM and 50 nM, and incubated for 48 and 72 hours. Lysates were collected, and the remaining

EXCLI Journal 2026;25:824-845 – ISSN 1611-2156
Received: March 05, 2026, accepted: May 22, 2026, published: June 19, 2026

expression of Rab11a and KIF13A proteins was measured using Western blotting. Above each Western blot, relative protein levels are presented for cells in which Rab11a and KIF13A were silenced using distinct siRNAs. β-tubulin was used as a loading control, and the relative expression of the proteins was normalised to the level of intracellular tubulin. A minimum of three independent experiments were conducted in total. Asterisks (*) indicate statistically significant differences ($p < 0.05$).

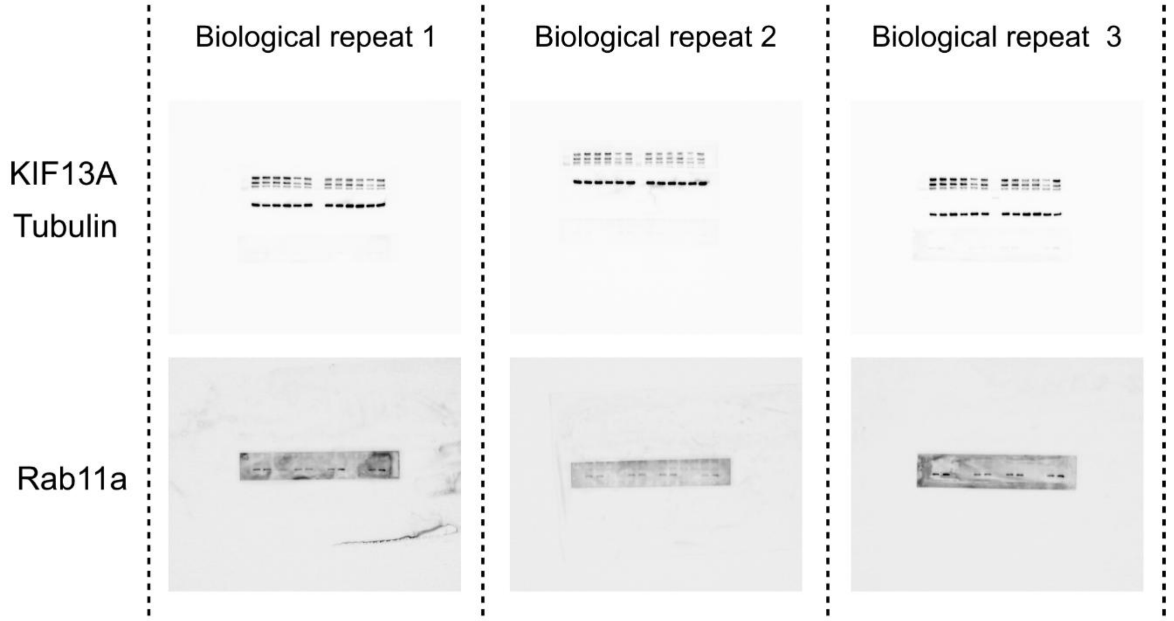

Supplementary Figure 3: Western blot images representing Rab11a and KIF13A knockdown by siRNA under different experimental conditions. The figure presents full-length Western blot images of KIF13A (at 250 kDa), beta tubulin loading control (at 55 kDa), and Rab11a (at 23 kDa) in control and Rab11a- or KIF13A-siRNA-treated cells across three biological replicates.

EXCLI Journal 2026;25:824-845 – ISSN 1611-2156
Received: March 05, 2026, accepted: May 22, 2026, published: June 19, 2026

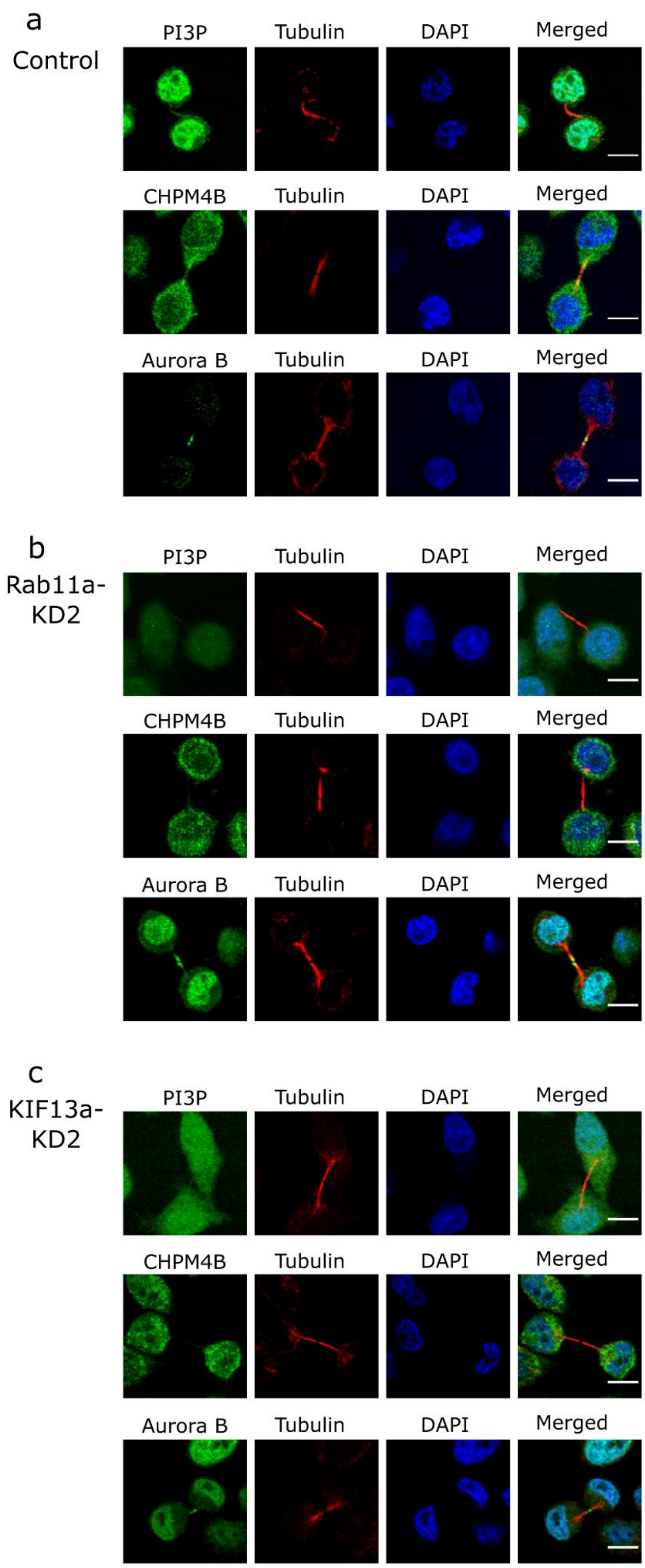

EXCLI Journal 2026;25:824-845 – ISSN 1611-2156
Received: March 05, 2026, accepted: May 22, 2026, published: June 19, 2026

Supplementary Figure 4: The interacting partners of KIF13A and its proposed cargo, Rab11a, localise to the EPs and the ICB during M-phase. (a-c) The localisation of putative downstream effectors of the KIF13A-Rab11a axis – namely CHMP4B, which is essential for cytokinesis completion; the abscission-delay regulator Aurora B; and PI3P, which acts as an anchor for FYVE-CENT – was examined in stable cell lines treated either with a non-targeting shRNA control or shRNAs specifically depleting Rab11a or KIF13A. Stable cell lines expressing different shRNAs were fixed and stained with DAPI, an anti-acetylated-α-tubulin antibody, and a specific antibody against PI3P, CHMP4B or Aurora B. The localisation pattern relative to tubulin at the ICB and the EPs was analysed in cytokinesis. Scale bar indicates 10 µm.

Supplementary Movie 1: Normal mitotic division of HeLa GFP-Tubulin mCherry-H2B cells treated with a non-targeting siRNA control.
For this experiment, HeLa GFP-Tubulin mCherry-H2B cells were treated with a non-targeting siRNA control for 48 h before imaging. Cells were imaged at 2 min intervals for 480 min with 1-µm z-plane slices, covering the entire mitotic spindle. All images show the maximum projections of z-stacks from representative data across at least 3 biological replicates per condition. Cell division duration was quantified by tracking the time spent from nuclear envelope breakdown to cytokinetic abscission. Scale bar indicates 10 µm.

Supplementary Movie 2: Prolonged mitotic division of HeLa GFP-Tubulin mCherry-H2B cells treated with KIF13A-siRNA1.
For this experiment, HeLa GFP-Tubulin mCherry-H2B cells were treated with KIF13A-siRNA1 for 48 h before imaging. Cells were imaged at 2 min intervals for 480 min with 1-µm z-plane slices, covering the entire mitotic spindle. All images show the maximum projections of z-stacks from representative data across at least 3 biological replicates per condition. Cell division duration was quantified by tracking the time spent from nuclear envelope breakdown to cytokinetic abscission. Scale bar indicates 10 µm.

EXCLI Journal 2026;25:824-845 – ISSN 1611-2156
Received: March 05, 2026, accepted: May 22, 2026, published: June 19, 2026

Supplementary experimental procedures

Antibodies and other staining reagents

Supplementary Table 1

Antigen	Source	Dilution	Company	Catalog #	Application
**Beta tubulin**	Mouse	1:3000	Invitrogen	MA5-16308	WB
**Alpha tubulin**	Mouse	1:10000	Merck	T5168	WB
**Rab11a**	Rabbit	1:250	Sigma	HPA051697	WB, IF
**KIF13A**	Rabbit	1:250	TFS	PA530874	WB, IF
**HRP reagent**	Mouse	1:9	TFS	35060	WB
**HRP reagent**	Rabbit	1:9	TFS	35061	WB
**Acetyl-α-tubulin**	Rabbit	1:400	Cell signalling	D20G3	IF
**Acetyl-α-tubulin**	Mouse	1:400	Invitrogen	32-2700	IF
**PI3P**	Mouse	1:250	Echelon Biosciences	Z-P003	IF
**CHMP4B**	Rabbit	1:250	Proteintech	13683-1-AP	IF
**Aurora B**	Mouse	1:200	TFS	MA515321	IF
**Rab11a**	Mouse	1:250	Nordic Biosite	ABB-FPO8IL-10	IF
**LAP2**	Rabbit	1:250	Invitrogen	PA5-52519	IF
**Alexa Flour 488**	Anti-mouse	1:200	Invitrogen	A21131	IF
**Alexa Flour 568**	Anti-rabbit	1:200	Abcam	AB175471	IF
**Alexa Flour 488**	Anti-mouse	1:200	TFS	A11001	IF
**Alexa Flour 488**	Anti-rabbit	1:200	Invitrogen	A11008	IF
**Alexa Fluor phalloidin 568**	N/A	1:50	TFS	A12380	IF
